# Supplementary material for: HIVprotI: an integrated web based platform for prediction and design of HIV proteins inhibitors
Source: J Cheminform. 2018 Mar 9;10:12. doi: 10.1186/s13321-018-0266-y (PMC5845081; doi:10.1186/s13321-018-0266-y)
Supplement: Supplementary file 2 — Additional file 2. Source code of HIVProtI web server. [file 13321_2018_266_MOESM2_ESM.zip › HIVprotI_Source-code/hivprotI/weka-3-6-6/doc/weka/core/pmml/FieldMetaInfo.Value.html]

FieldMetaInfo.Value


---


|  |  |  |
| --- | --- | --- |
| **Overview** \| **Package** \| **Class** \| **Tree** \| **Deprecated** \| **Index** \| **Help** \| **Weka's home** | |  |
| **PREV CLASS**   **NEXT CLASS** | **FRAMES**    **NO FRAMES**     **All Classes** |  |
| SUMMARY: NESTED \| FIELD \| CONSTR \| METHOD | DETAIL: FIELD \| CONSTR \| METHOD |  |


---


## weka.core.pmml Class FieldMetaInfo.Value

```
java.lang.Object
  weka.core.pmml.FieldMetaInfo.Value
```

**All Implemented Interfaces:**: java.io.Serializable

**Enclosing class:**: FieldMetaInfo

---

``` public static class FieldMetaInfo.Value extends java.lang.Object implements java.io.Serializable ```

Inner class for Values

**See Also:**: Serialized Form

---

| **Nested Class Summary** | |
| --- | --- |
| `static class` | `FieldMetaInfo.Value.Property`             Enumerated type for the property. |


| **Method Summary** | |
| --- | --- |
| `java.lang.String` | `getDisplayValue()` |
| `FieldMetaInfo.Value.Property` | `getProperty()` |
| `java.lang.String` | `getValue()` |
| `java.lang.String` | `toString()` |

**Methods inherited from class java.lang.Object**

`equals, getClass, hashCode, notify, notifyAll, wait, wait, wait`

**Method Detail**

### toString

```
public java.lang.String toString()
```

:   **Overrides:**: `toString` in class `java.lang.Object`

---


### getValue

```
public java.lang.String getValue()
```

---


### getDisplayValue

```
public java.lang.String getDisplayValue()
```

---


### getProperty

```
public FieldMetaInfo.Value.Property getProperty()
```


---


|  |  |  |
| --- | --- | --- |
| **Overview** \| **Package** \| **Class** \| **Tree** \| **Deprecated** \| **Index** \| **Help** \| **Weka's home** | |  |
| **PREV CLASS**   **NEXT CLASS** | **FRAMES**    **NO FRAMES**     **All Classes** |  |
| SUMMARY: NESTED \| FIELD \| CONSTR \| METHOD | DETAIL: FIELD \| CONSTR \| METHOD |  |


---
